# Ongoing clinical trials of PD-1 and PD-L1 inhibitors for lung cancer in China

**DOI:** 10.1186/s13045-017-0506-z

**Published:** 2017-07-05

**Authors:** Si-Yang Liu, Yi-Long Wu

**Affiliations:** Guangdong Lung Cancer Institute, Guangdong General Hospital & Guangdong Academy of Medical Sciences and School of Medicine of South China University of Technology, 106 Zhongshan 2nd Road, Guangzhou, 510080 China

**Keywords:** PD-1 inhibitor, PD-L1 inhibitor, Clinical trials, China

## Abstract

Compared to chemotherapy, promising results have been obtained by blocking the PD-1 pathway using antibodies that inhibit programmed cell death protein 1 (PD-1) or programmed cell death protein ligand 1 (PD-L1). Furthermore, global researchers and doctors are exploring how to optimize this immunotherapy in 270 clinical studies. However, Chinese clinical trials of these agents remain in the early stages. We summarize the ongoing international and domestic clinical trials using PD-1 and PD-L1 inhibitors to treat lung cancer. This information can help researchers better understand the active and approved clinical trials in China, as well as the ongoing research regarding PD-1 and PD-L1 inhibitors.

## Background

Immunotherapy is revolutionizing the treatment of multiple cancer types and provides promising clinical responses, durable disease control, and fewer adverse events among patients with advanced melanoma, non-small cell lung cancer (NSCLC), and other tumor types [1]. Programmed cell death protein-1 (PD-1) is an important immune checkpoint protein that is expressing on the surface of T cells. When PD-1 binds to its ligands, such as programmed cell death protein ligand 1 (PD-L1) and programmed cell death protein ligand 2, T cell activity and cytokine production is greatly downregulated at the tumor site. This effect contrasts with the biological characteristics of treatments targeting cytotoxic T lymphocyte-associated protein 4 (CTLA-4), and PD-1/PD-L1 blockage is now considered a “tumor site immune modulation therapy” [2, 3]. Blocking the PD-1 pathway using monoclonal antibodies to PD-1 or PD-L1 can stimulate the patient’s immune system to kill tumor cells, and this approach has provided remarkable anti-tumor efficacy, compared to standard first-line and second-line chemotherapy for advanced NSCLC [4–8]. By the end of December 2016, the US Food and Drug Administration had approved PD-1 pathway blockade treatments using nivolumab, pembrolizumab, and atezolizumab [9].

In China, no PD-1 or PD-L1 inhibitors have received marketing approval from the Chinese Food and Drug Administration (CFDA). However, various clinical trials are actively investigating international and domestic drugs. Between January 1, 2013 and April 6, 2017, ClinicalTrials.gov registered 270 international clinical trials using PD-1/PD-L1 therapies for NSCLC (e.g., nivolumab, pembrolizumab, atezolizumab, and durvalumab). These 270 trials included 61 studies that involved East Asian sites and 14 studies that involved Chinese sites (12 multinational trials and 2 trials that only evaluated Chinese patients). Thus, research regarding immune checkpoint inhibitors in China is several years behind research in other areas of the world. This review evaluates the ongoing international and domestic clinical trials using PD-1 or PD-L1 inhibitors, and we hope this review will provide detailed information regarding how to perform immunotherapy clinical trials in China.

## International trials

**Fig. 1:**
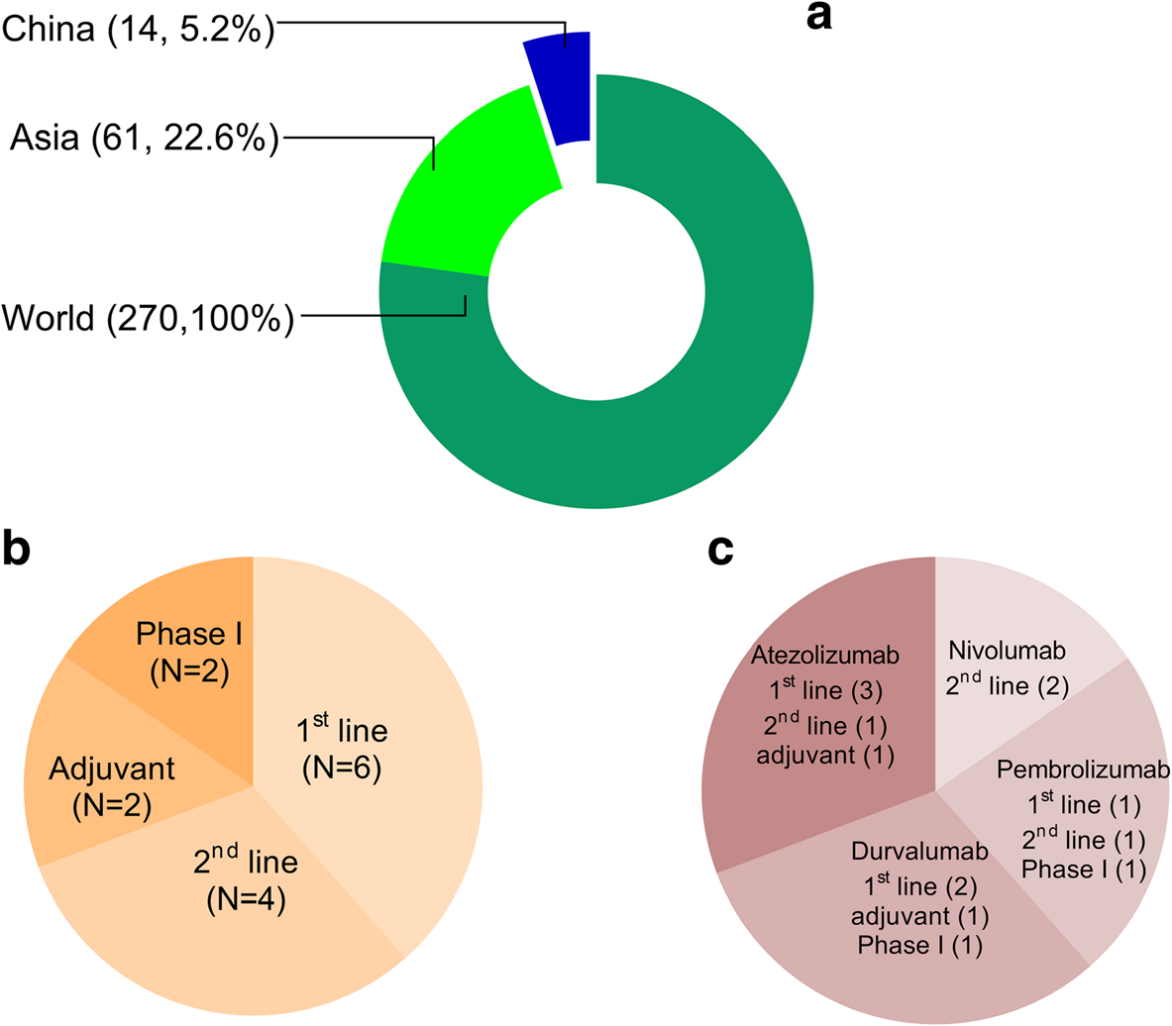
Ongoing international clinical trials according to whether they included Chinese patients. **a** Between January 1, 2013, and April 6, 2017, there were 270 clinical trials of anti-PD-1/PD-L1 inhibitors for NSCLC that were registered on ClinicalTrials.gov. Among the 270 studies, 61 studies were performed in East Asia and 14 studies were performed in China (12 multinational trials and 2 trials that only evaluated Chinese patients). **b** The 14 clinical trials included six first-line studies, four second-line studies, two adjuvant therapy studies, and two phase I studies for only Chinese patients. **c** The classification of clinical trials in China according to the therapeutic agent, which includes nivolumab, pembrolizumab, atezolizumab, and durvalumab

Chinese participation in international multicenter clinical trials using PD-1/PD-L1 inhibitors has greatly increased during the last 3 years. Based on our research, Chinese sites were involved in 14 global clinical trials by April 6, 2017. These trials included six first-line trials (NCT02220894 [KEYNOTE 042], NCT03003962, NCT02542293 [NEPTUNE], NCT02763579 [IMpower133], NCT02657434 [IMpower132], and NCT02409342 [IMpower110]), four second-line trials (NCT02864394 [MK-3475-033], NCT02613507 [CheckMate078], NCT02481830 [CheckMate331], and NCT02813785 [IMpower210]), two adjuvant therapy trials (NCT02486718 [IMpower010] and NCT02273375 [BR.31]), and two trials that only evaluated Chinese patients (NCT02835690 [KEYNOTE 032] and NCT02978482) (Fig. 1).

### First-line

#### Pembrolizumab (KEYNOTE 042)

KEYNOTE 042 was an international, open-label, randomized, phase III study that evaluated overall survival (OS) among treatment-naïve patients who received pembrolizumab (MK-3475) or platinum doublet chemotherapy for advanced or metastatic NSCLC. The KEYNOTE 024 trial enrolled patients with high PD-L1 expression (proportion score of ≥50%), while the KEYNOTE 042 trial evaluated patients with a PD-L1 tumor proportion score of ≥1% based on immunohistochemistry. Patients were excluded if their tumors harbored sensitive mutations in the epidermal growth factor receptor (*EGFR*) gene or translocation of the anaplastic lymphoma kinase (*ALK*) gene. Participants were randomized 1:1 to receive either pembrolizumab monotherapy (200 mg every 3 weeks for up to 35 treatments or until disease progression) or carboplatin plus pemetrexed or paclitaxel (non-squamous tumor histology) for 4–6 cycles. The primary study hypothesis was that pembrolizumab would prolong OS compared to the chemotherapeutic standard of care (SoC). Thus, the primary endpoint was defined as OS and the secondary endpoint was defined as progression-free survival (PFS), which was evaluated by a central independent radiologist. This trial closed during February 2017 in China.

#### Durvalumab (NCT03003962)

The NCT03003962 trial is an open-label, multicenter, phase III study that is focused on Asian patients. Chinese hospitals account for 50% of the centers (16/32), and the study aims to enroll 440 patients with stage IV NSCLC, wild-type *EGFR* and *ALK*, and high PD-L1 expression (proportion score of ≥25%). These patients are randomized 1:1 to receive first-line treatment using either durvalumab (MEDI4736, a PD-1 inhibitor) or SoC platinum-based chemotherapy. The two primary objectives are to assess the treatment efficacies based on PFS and OS. The trial enrolled the first Chinese patients in February 2017 and will close in 2018.

#### Durvalumab (NEPTUNE)

Tremelimumab is an anti-CTLA4 antibody that may provide clinical benefits in patients with melanoma and some other tumors. However, it remains unclear whether a combination of two checkpoint inhibitors with completely different biological mechanisms can increase the patient response rates [2, 10]. The results of a phase I study using durvalumab (20 mg/kg every 4 weeks) plus tremelimumab (1 mg/kg) have been published in the *Lancet Oncology* [11] and revealed that this treatment provided manageable tolerability and anti-tumor activity, regardless of PD-L1 status. The NEPTUNE study is a global phase III study that compares platinum-based SoC chemotherapy to durvalumab plus tremelimumab immunotherapy for untreated patients with advanced or metastatic NSCLC and wild-type *EGFR* and *ALK*, regardless of their PD-L1 expression. Compared to the platinum-based SoC chemotherapy, the efficacy and safety of durvalumab will be evaluated in terms of OS. Patients are randomized 1:1 to the two treatment arms. The trial is currently recruiting patients and enrolled its first Chinese patient on February 4, 2017. The results will be released in 2018.

#### Atezolizumab (IMpower110)

The IMpower110 trial compares platinum plus pemetrexed (non-squamous disease) or gemcitabine (squamous disease) versus atezolizumab (MPDL3280A) among chemotherapy-naïve patients with stage IV NSCLC. The study considers both safety and efficacy outcomes. Patients with previously detected sensitizing *EGFR* mutations or *ALK* fusions may have received tyrosine kinase inhibitor treatment. Positive PD-L1 expression is determined using immunohistochemistry and a proportion score of ≥1%. This trial has not started recruiting patients in China.

#### Atezolizumab (IMpower133)

The clinical benefits and toxicities of combination immunotherapy for small cell lung cancer (SCLC) remain unclear, and several international clinical trials are ongoing [12]. The IMpower133 trial is a phase I/III, multicenter, double-blinded, placebo-controlled study that evaluates the safety and efficacy of atezolizumab (a PD-L1 inhibitor) with or without carboplatin plus etoposide. This study evaluates treatment-naïve patients with extensive-stage SCLC, who are randomized 1:1 to the two treatment arms. The induction phase is four 21-day cycles, and maintenance treatment using atezolizumab or placebo is provided until persistent radiographic disease progression or symptomatic deterioration is detected. This trial is currently recruiting patients in China and enrolled its first patient on April 2017. The estimated completion date is July 31, 2019.

#### Atezolizumab (IMpower132)

The IMpower132 trial is a randomized, phase III, multicenter, open-label study of patients who are chemotherapy-naïve and have stage IV non-squamous NSCLC. Patients are excluded if they have received prior treatment, a sensitizing EGFR mutation, or an ALK fusion oncogene. The primary outcome measures are PFS and OS to evaluate the safety and efficacy of atezolizumab in combination with cisplatin or carboplatin plus pemetrexed, and eligible patients will be randomized 1:1 into arm A (atezolizumab + carboplatin or cisplatin + pemetrexed) and or arm B (carboplatin or cisplatin + pemetrexed). The trial has not started recruiting patients (Table 1).

**Table 1:** Ongoing international clinical trials of PD-1 and PD-L1 inhibitors on lung cancer with participating Chinese centers

Indentifier	Drugs	Location(s)	Phase	Indication	Population	Status in China
NCT02220894 KEYNOTE 042	Pembrolizumab	International	III	First-line	Advanced or metastatic NSCLC, *EGFR* and *ALK* wild-type, PD-L1-positive	Closed
NCT03003962 −	Durvalumab	Asia (16/32)	III	First-line	Advanced NSCLC, *EGFR* and *ALK* wild-type, PD-L1-high expression	Recruiting
NCT02542293 NEPTUNE	Durvalumab D + T	International	III	First-line	Advanced or metastatic, NSCLC, *EGFR* and *ALK* wild-type	Recruiting
NCT02409342 IMpower110	Atezolizumab	International	III	First-line	Chemotherapy-naïve and stage IV NSCLC, PD-L1-positive	Not yet recruiting
NCT02763579 IMpower133	Atezolizumab A + C + E	International	I/III	First-line	Extensive-stage SCLC	Recruiting
NCT02657434 IMpower132	Atezolizumab A + C + P	International	III	First-line	Chemotherapy-naïve and stage IV non-squamous NSCLC	Not yet recruiting
NCT02613507 CheckMate078	Nivolumab	Asia (23/32)	III	Second-line and beyond	Stage IIIB/IV or recurrent NSCLC after failure with platinum-containing doublet chemotherapy	Closed
NCT02864394 MK-3475-033	Pembrolizumab	International	III	Second-line and beyond	Stage IIIB/IV or recurrent NSCLC after failure with platinum-containing chemotherapy, PD-L1-positive, no *EGFR sensitizing mutation*	Recruiting
NCT02481830 CheckMate331	Nivolumab	International	III	Second-line	Relapsed SCLC after platinum-based first-line chemotherapy	Not yet recruiting
NCT02813785 IMpower 210	Atezolizumab	Asia (27/40)	III	Second-line and beyond	NSCLC after failure with platinum-containing chemotherapy	Recruiting
NCT02486718 IMpower 010	Atezolizumab	International	III	Adjuvant therapy	Stage IB–IIIA NSCLC following resection and adjuvant chemotherapy	Recruiting
NCT02273375 BR.31	Durvalumab	International	III	Adjuvant therapy	Completely resected stage IB–IIIA NSCLC	Recruiting
NCT02835690 KEYNOTE 032	Pembrolizumab	China	I	–	Locally advanced or metastatic NSCLC	Not yet recruiting
NCT02978482 –	Durvalumab D + T	China	I	–	Advanced malignancies	Recruiting

*NSCLC* non-small cell lung cancer, *EGFR* epidermal growth factor receptor, *ALK* anaplastic lymphoma kinase, *PD-L1* programmed cell death protein ligand 1, *D + T* durvalumab plus tremelimumab, *A + C + E* atezolizumab plus carboplatin plus etoposide

### Second-line

#### Nivolumab (CheckMate078)

The Checkmate078 trial is the first Chinese study of a PD-1 inhibitor. This trial focused on Asian patients and aimed to enroll 500 patients with advanced or metastatic NSCLC who had failed platinum-based doublet chemotherapy. Chinese hospitals accounted for >70% of the centers (23/32). Patients were randomized 2:1 to receive either nivolumab (MDX-1106; 3 mg/kg every 2 weeks) or docetaxel (75 mg/m^2^ every 3 weeks). Patients with *EGFR* mutations were excluded, and *ALK* status was not considered. The primary outcome measure was OS. The trial has completed its recruitment, and the results will be released during 2017.

#### Pembrolizumab (MK-3475-033)

The study aims to assess the efficacies of pembrolizumab versus docetaxel among patients with stage IIIB/IV or recurrent NSCLC who have experienced disease progression after platinum-containing systemic therapy. *Patients may not have an EGFR sensitizing mutation and must have positive PD-L1 expression (tumor proportion score of ≥1%) based on immunohistochemistry.* The primary hypothesis of the study is that pembrolizumab will prolong OS and PFS, compared to docetaxel, among participants with PD-L1-positive tumors. The trial is recruiting patients in China, and the estimated study completion date is January 28, 2019.

#### Nivolumab (CheckMate 331)

Topotecan has been approved as a second-line treatment for SCLC but has provided disappointing efficacy. A phase I/II trial (CheckMate 032) revealed promising results from dual blockade of PD-1 and CTLA-4 for relapsed SCLC, compared with topotecan [13]. Thus, the phase III Checkmate 331 study compares nivolumab versus chemotherapy among patients with relapsed SCLC after failed platinum-based first-line chemotherapy. Patients are randomized to three treatment arms: the experimental arm with nivolumab, an active comparator arm with topotecan, and a second active comparator arm with amrubicin. Data from the ongoing phase III trial are needed to confirm whether nivolumab is effective for treating SCLC. This clinical trial has not started recruiting patients in China.

#### Atezolizumab (IMpower 210)

The IMpower 210 study is a multicenter, open-label, randomized controlled phase III study with sites in five East Asian countries. This study aims to evaluate the efficacy and safety of atezolizumab versus docetaxel among patients with locally advanced or metastatic NSCLC that has progressed during or after platinum-containing treatment. The study’s treatment will continue until disease progression or unacceptable toxicity is detected. The estimated enrollment is 563 patients, and the design targets 80% of the enrolled patients being recruited from 27 Chinese hospitals. Atezolizumab is administered at a fixed dose of 1200 mg using intravenous (IV) infusion on day 1 of each 21-day cycle. Docetaxel is administered as an IV infusion at a dose of 75 mg/m^2^ on day 1 of each 21-day cycle. The trial is currently recruiting patients in China, and the estimated study completion date is May 31, 2019.

### Adjuvant therapy

#### Atezolizumab (IMpower 010)

The IMpower 010 study is a phase III, global, multicenter, open-label, randomized study to compare the efficacy and safety of 16 atezolizumab treatment cycles, compared to best supportive care, for patients with stage IB–IIIA NSCLC after resection and adjuvant chemotherapy. Participants will complete ≤4 cycles of adjuvant cisplatin-based chemotherapy and will then be randomized 1:1 to receive 16 cycles of atezolizumab treatment or best supportive care. The primary outcome is disease free survival (DFS), and the secondary outcome is OS. The IMpower010 study is currently recruiting patients in China, and the estimated study completion date is September 25, 2026.

#### Durvalumab (BR.31)

The durvalumab study (NCT02273375) is a phase III, prospective, double-blind, placebo-controlled, randomized study of patients with completely resected NSCLC. The study aims to compare durvalumab to chemotherapy after surgery. The primary outcome measure is DFS, and all patients must have NSCLC with positive PD-L1 expression. This study is sponsored by the Canadian Cancer Trials Group, and BR.31 is an international intergroup trial that involves the Chinese Thoracic Oncology Group. This trial is currently recruiting patients in China, and the estimated study completion date is January 2025.

### Clinical trials for only Chinese patients

#### Pembrolizumab (KEYNOTE 032) and durvalumab (NCT02978482)

There are no available clinical data regarding pembrolizumab and durvalumab among Chinese patients. The phase I trials were launched in August 2016 and December 2016 and aim to assess the safety, tolerability, pharmacokinetics, and efficacy of these agents among Chinese patients. The KEYNOTE 032 study focuses on pembrolizumab monotherapy for patients with locally advanced or metastatic NSCLC. The durvalumab study evaluates the combination of durvalumab plus tremelimumab for patients with advanced malignancies. KEYNOTE 032 has not started recruiting patients in China. The durvalumab trial (NCT02978482) is currently recruiting patients in China, and the estimated study completion date is June 28, 2018.

## Domestic clinical trials

**Fig. 2:**
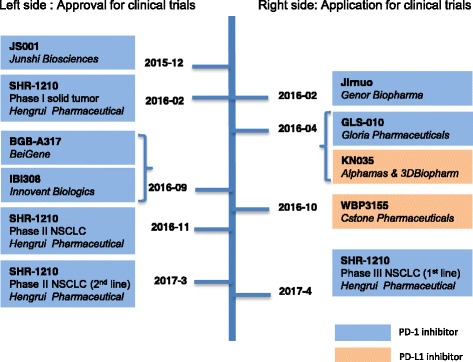
The development process of eight national anti-PD1 and PD-L1 inhibitors. The agents on the *left* were approved by the Chinese Food and Drug Administration for clinical trials, and the agents on the *right* have applied for clinical trial approval. *Blue rectangles* indicate PD-1 inhibitors, and *orange rectangles* indicate PD-L1 inhibitors

Significant efforts by the Chinese government during the last 2 years have led to improvements in the overall clinical research environment [14]. Now the CFDA is actively reforming the regulatory framework for the approval of novel agents, which has introduced a “four-color light” strategy and will likely further adjust its policies to reflect advances in large international clinical trials of immunotherapy [15]. Chinese pharmaceutical companies had developed eight anti-PD-1/PD-L1 inhibitors by January 7, 2017, and four drugs (JS001, SHR-1210, IBI308, and BGB-A317) have been approved by the CFDA for phase I trials among patients with advanced solid tumors and NSCLC. Another four drugs (Jirnuo monoclonal antibodies, GLS-010, KN035, and WBP3155) are now being considered by the CFDA for clinical trial approval (Fig. 2). In November 2016, th﻿e ﻿PD-1 inhibitor KN035, ​that is administered by subcutaneous injection, received approval from the American Food and Drug Administration for clinical trial conduction.

### SHR-1210 (CTR20160175 and CTR20170090)

SHR1210 is an anti-PD-1 checkpoint inhibitor that was developed by Shanghai Hengrui Pharmaceutical Co. Ltd. The CTR20160175 trial is estimated to enroll 32–51 Chinese patients with advanced solid tumors (e.g., lung cancer, nasopharyngeal carcinoma, esophagus carcinoma, gastric carcinoma, and hepatic carcinoma). The main purpose of this study is to evaluate the drug’s safety and tolerability, which would lay the foundation for research regarding the drug’s delivery mode. The CTR20170090 trial is investigating a combination of SHR-1210 and apatinib, which is a first-generation oral anti-angiogenesis drug. The main objective of the phase II study is to evaluate the safety and tolerability of this combination, and a phase III trial is planned. Both trials are currently recruiting patients in China and enrolled their first patients on April 6, 2016, and March 21, 2017, respectively.

### SHR-1210 (CTR20170299 and CTR20170322)

The CTR20170090 trial is a phase II, open-label, single-arm, multicenter study of patients with stage IIIB/IV NSCLC who have failed platinum-based chemotherapy. The study aims to investigate the objective response rate for SHR-1210 among patients with varying PD-L1 expressions (proportion score of ≥50%). Patients with *EGFR* mutations or *ALK* fusions may have received tyrosine kinase inhibitor treatment. The CTR20170322 trial is a phase III, open-label, randomized multicenter study that compares SHR-1210 plus pemetrexed and carboplatin versus pemetrexed plus carboplatin for patients with stage IIIB/IV non-squamous NSCLC. Patients have wild-type *EGFR* and *ALK*, and PD-L1 expression is not considered. The primary endpoint is PFS. Both trials have not started recruiting patients in China.

### JS-001 (CTR20160274)

JS-001 is a recombinant humanized monoclonal antibody to PD-1 that was developed by Shanghai Junshi Biosciences Co., Ltd. This drug was the first PD-1 inhibitor to be approved for clinical trials by the CFDA. The CTR20160274 trial is a phase I, single-arm, non-randomized study that aims to evaluate the safety, tolerability, and antitumor activity of the drug among Chinese patients with advanced or recurrent malignant tumors. The expression of PD-L1 is not mentioned in the study’s protocol. This clinical trial is currently recruiting patients in China.

### IBI308 (CTR20160735)

IBI308 is a powerful antibody to PD-1 that was developed by Innovent Biologics. Compared to other drugs, IBI308 has a higher affinity for PD-1 and induces PD-1 endocytosis to activate T cells. Preclinical data suggest that the drug provides good anti-tumor efficacy, and the CTR20160735 trial is a phase I study that is evaluating the safety, tolerability, and antitumor activity of IBI308 alone or in combination with chemotherapy among Chinese patients with malignant tumors (e.g., lung carcinoma, hepatic carcinoma, and melanoma). The primary outcome is the therapeutic efficacy of the two IBI308-containing regimens, based on the immune-related Response Evaluation Criteria. The study also aims to identify potential prognostic biomarkers using tumor tissues from patients who experienced a curative effect. This clinical trial is currently recruiting patients in China and enrolled its first patient on October 24, 2016.

### BGB-A317 (CTR20160872)

BGB-A317 has been specifically designed to block the binding activity of the Fc-gamma receptor I. At the 2016 meeting of the American Society of Clinical Oncology, BeiGene reported preliminary clinical data from an Australian phase I study of patients with 26 different types of tumors (although the tumors did not include lung cancer or melanoma). The CTR20160872 trial is a phase I study that aims to assess the safety, tolerability, pharmacokinetics, and antitumor activity of BGB-A317 among Chinese patients with advanced solid tumors, such as lung carcinoma, hepatic carcinoma, and gastric carcinoma. A phase III study will be launched in 2017 and will incorporate PD-L1 testing using immunohistochemistry. This clinical trial has started recruiting patients in China (Table 2).

**Table 2:** Ongoing clinical trials with domestic PD-1 or PD-L1 inhibitors on advanced tumors and NSCLC

Drugs and indentifier	The role of China	Phase	Indication	Design	State	Enrollment
SHR-1210 CTR20160175	Sponsor	I	Advanced solid tumors	Non-randomized, open-label, single-center, dose-escalation	Recruiting	45
SHR-1210 CTR20170090	Sponsor	II S + A	Advanced NSCLC	Non-randomized, open-label, single-center, dose-escalation	Recruiting	118
SHR-1210 CTR20170299	Sponsor	II	Advanced NSCLC	Open-label, single-arm, multicenter	Not yet recruiting	120
SHR-1210 CTR20170322	Sponsor	III S + P + C	Advanced non-squamous NSCLC	Randomized, open-label, multicenter	Not yet recruiting	412
JS-001 CTR20160274	Sponsor	I	Advanced solid tumors	Open-label, single-group assignment	Recruiting	54
IBI308 CTR20160735	Sponsor	I	Advanced malignant tumors	Non-randomized, open-label, single-group assignment	Recruiting	104
BGB-A317 CTR20160872	Sponsor	I	Advanced solid tumors	Non-randomized, open-label, single-group assignment	Not yet recruiting	300

*NSCLC* non-small cell lung cancer, *S + A* SHR-1211 plus apatinib, *S + P + C* SHR-1210 plus pemetrexed and carboplatin

## Discussion

Immunotherapy using PD-1 or PD-L1 inhibitors provides durable clinical regression, good tolerance, and relatively few adverse events among patients with malignant tumors. However, Chinese patients have limited access to these promising immune-checkpoint inhibitors, such as antibodies to PD-1 and PD-L1. Furthermore, the ongoing international and domestic clinical trials have duplicated the designs of previous trials for PD-1 and PD-L1 antibodies, which have revealed promising results, but rarely consider the characteristics of the Chinese or Asian populations.

The characteristics of Chinese patients are important considerations in trial designs, as the Chinese population has relatively high rates of hepatitis B virus infection and traditional medicine use [16]. Thus, many Chinese patients are ineligible for clinical trials of the antibodies to PD-1 or PD-L1. Furthermore, Chinese and international researchers have revealed that Chinese patients with NSCLC may have more driver gene mutations, different gene profiles, better clinical responses to chemotherapy, and different toxicity profiles [17–20]. However, the relevance of these characteristics remains unclear for immunotherapy with blockade of the PD-1 pathway, and data from Chinese patients remain scarce.

At the 14th Lung Cancer Summit, Chinese scientists introduced several proposals for immunotherapy using PD-1 or PD-L1 inhibitors. First, the much higher proportion of *EGFR* mutation should be considered when designing Chinese immunotherapy trials, as the prevalence of *EGFR* mutations is approximately 50% among Chinese patients with NSCLC [21, 22]. Second, the introduction of four FDA-approved immunohistochemistry biomarker assays, and the development of domestic reagents, may allow for the development of PD-1 immunohistochemistry assays in China. A phase II single-arm study of SHR-1210 as a second-line NSCLC treatment is aiming to determine an optimal PD-L1 cut-off value for Chinese patients.

Global researchers remain faced with unanswered questions regarding PD-1 and PD-L1 inhibitors. First, the relationships between PD-L1 expression and other factors (e.g., mutational burden, smoking index, and neoantigen quantity) are very complicated [23, 24], and further research is needed. Second, it would be useful to develop combined biomarkers and complementary assays that could improve the overall specificity (58%) and sensitivity (72%) of PD-1 expression [1, 25]. Third, it is possible that combining immunotherapy with chemotherapy, targeted therapy, and radiotherapy could provide superior results, compared to immunotherapy alone. Fourth, although we have known that antibodies to PD-L1 have relatively low pulmonary toxicity, it remains unclear whether antibodies to PD-1 or PD-L1 provide a better clinical response. Fifth, the conventional Response Evaluation Criteria In Solid Tumors are not appropriate for evaluating PFS after immunotherapy and pseudo-progression, and additional research is needed to improve the utility of immune-related response criteria [26].

## Conclusions

Chinese clinical trials using PD-1 or PD-L1 inhibitors remain in their early stages, and further efforts are needed to improve the design of future clinical trials. In addition, China must take steps to help translate the results of clinical trials into clinical practice. For example, it will be useful to simplify phase I study designs and speed up late-stage clinical development.

## Abbreviations

ALK: Anaplastic lymphoma kinase
CFDA: Chinese Food and Drug Administration
CTLA-4: Cytotoxic T lymphocyte-associated protein 4
DFS: Disease-free survival
EGFR: Epidermal growth factor receptor
NSCLC: Non-small cell lung cancer
OS: Overall survival
PD-1: Programmed cell death protein-1
PD-L1: Programmed cell death protein ligand 1
PFS: Progression-free survival
SCLC: Small cell lung cancer
SoC: Standard of care
